# Is heart transplantation a valuable option in patients with diffuse systemic sclerosis and primary cardiac involvement?

**DOI:** 10.1002/ccr3.2600

**Published:** 2019-12-19

**Authors:** Frédéric Turcotte‐Gosselin, Pierre Yves Turgeon, Alena Ikic, Jonathan Beaudoin, Philippe Joubert, Michelle Dubois, Pierre Voisine, Mario Sénéchal

**Affiliations:** ^1^ Department of Cardiology Institut Universitaire de Cardiologie et de Pneumologie de Québec Laval University Quebec QC Canada; ^2^ Department of Rheumatology CHUL‐CHU of Quebec Laval University Quebec QC Canada; ^3^ Department of Anatomopathology Institut Universitaire de Cardiologie et de Pneumologie de Québec Laval University Quebec QC Canada; ^4^ Research Center Institut Universitaire de Cardiologie et de Pneumologie de Québec Laval University Quebec QC Canada; ^5^ Department of Cardiac Surgery Institut Universitaire de Cardiologie et de Pneumologie de Québec Laval University Quebec QC Canada

**Keywords:** cardiac magnetic resonance imaging, heart failure, heart transplantation, systemic sclerosis

## Abstract

Systemic sclerosis patients with primary cardiac involvement can be reliably diagnosed by cardiac magnetic resonance imaging and are associated with a poor prognosis. This case report highlights the importance of considering heart transplantation in those patients as a lifesaving procedure.

## Graphical abstract

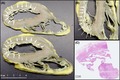

## 1 INTRODUCTION

Systemic sclerosis (SSc) is a multisystemic disorder manifested by autoimmune and fibrotic changes involving the skin and internal organs. Whereas lung transplantation has been widely reported in SSc, heart transplantation in an adult patient has been rarely described. We report a case of a female patient with SSc with predominant cardiac involvement characterized by severe subendocardial fibrosis as evaluated by preoperative cardiac magnetic resonance imaging (CMR). We discuss the specific pretransplant evaluation in patient with SSc and the possible role of CMR in the evaluation of cardiac fibrosis.

Systemic sclerosis is a rare multisystem connective tissue disease characterized by extensive skin thickening and multiorgan involvement.^1^ Systemic sclerosis may affect the myocardium with myocardial fibrosis resulting from multiple local ischemic lesions.^2^ Myocardial involvement is a common histological finding, but rarely causes severe left ventricular systolic dysfunction (approximately ≤10% of the patients).^2^ In general, myocardial fibrosis is considered to be the hallmark of cardiac involvement in SSc.^3^ The fibrosis tends to be patchy but distributed throughout the myocardium in both ventricles.^3^ Usually, the fibrosis involves the immediate subendocardial layer.^3^ In presence of severe systolic heart failure and diffuse endomyocardial fibrosis secondary to SSc and given the usual multiorgan involvement of the disease, the clinician may be reluctant to refer SSc patients for heart transplantation evaluation. We report a case of a patient with SSc with predominant heart involvement who underwent successful heart transplantation. We underscore the importance of ruling out any other significant organ involvement, as well as the role of cardiac magnetic resonance imaging (CMR) in the quantification of the burden of myocardial fibrosis, as a part of the pretransplantation evaluation.

## 2 CASE REPORT

**Figure 1:**
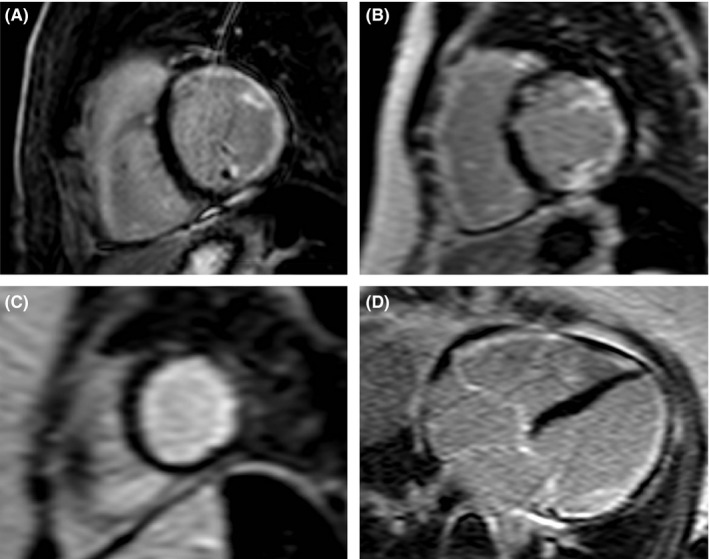
CMR late gadolinium enhancement (LGE) images in the basal (A), mid (B) and apical (C) short‐axis views and 4‐chamber view (D). There is subendocardial enhancement in the lateral LV wall, becoming circumferential at the apex. LGE is also seen in the right ventricle

A 48‐year‐old woman was hospitalized with symptoms of heart failure compatible with New York Heart Association class III; she was short of breath at minimal activity. Six years before, a diagnosis of diffuse SSc had been established on the basis of typical skin manifestations. The patient had an extensive skin induration proximal to the elbows and knees with truncal involvement and sclerodactyly. The modified Rodnan skin score (MRSS) was 28 out of 51 at diagnosis. She also had multiple telangiectasia and digital calcinosis. The antinuclear antibodies titer was 1/80 with speckled pattern. No specific antibody was identified after testing for anti‐dsDNA, anticentromere, anti‐Scl70, anti‐RNP, anti‐Jo1, anti‐Sm, anti‐SSA, and anti‐SSB. The patient was also tested for rare SSc antibodies that became negative. However, the patient was already on immunosuppressive therapy when the tests were done. The patient was diagnosed with idiopathic dilated cardiomyopathy a couple of years before the diagnosis of SSc. At that time, the LVEF was estimated at 35% with global mild hypokinesia and marked hypokinesia in the inferior territory. Coronary disease was excluded with angiography. The dosage of NTproBNP was chronically elevated at 844 ng/L but troponin T and CK levels were always normal. After few years, severe heart failure symptoms refractory to optimal guidelines directed therapy led to heart transplantation evaluation. At that time, the patient was under medical therapy consisting of furosemide (160 mg PO twice daily), spironolactone (25 mg PO daily), valsartan (80 mg PO daily), and bisoprolol (5 mg PO daily). Because of progressive fatigue and dyspnea attributed to low cardiac output, biweekly intravenous perfusion of milrinone (0.375 μg/kg) was begun 6 months before transplantation. Resting EKG showed sinus rhythm with incomplete right bundle branch block and nonspecific T‐waves repolarization anomaly. Prolonged cardiac monitoring was uneventful except for rare monomorphic premature ventricular contraction. Control transthoracic echocardiography revealed global hypokinesia with an ejection fraction of 25% and severe right ventricular dysfunction. A severe tricuspid regurgitation and mild to moderate mitral regurgitation were also present. A right‐side cardiac catheterization was done and demonstrated a pulmonary pressure of 17/10 mm Hg, and a mean pulmonary pressure of 14 mm Hg with a wedge pressure of 6 mm Hg. Calculated pulmonary resistance was evaluated at 2.9 woods unit and the transpulmonary gradient was 8 mm Hg. The cardiac output was considerably reduced at 2.8 L/min and with the corresponding cardiac index of 1.6 L/min/m^2^. CMR showed hypokinesis and dilatation of the right ventricle. In addition, right ventricle function was decreased to an ejection fraction of 25%. The left ventricle was moderately dilated to 125 mL/m^2^ with severe global hypokinesis. The CMR also demonstrated diffuse subendocardial late gadolinium enhancement compatible with fibrosis secondary to SSc (Figure 1). This fibrosis pattern was categorized as clearly nonischemic and would have been atypical for a myocarditis sequela; therefore, the patient did not underwent endomyocardial biopsy.

**Figure 2:**
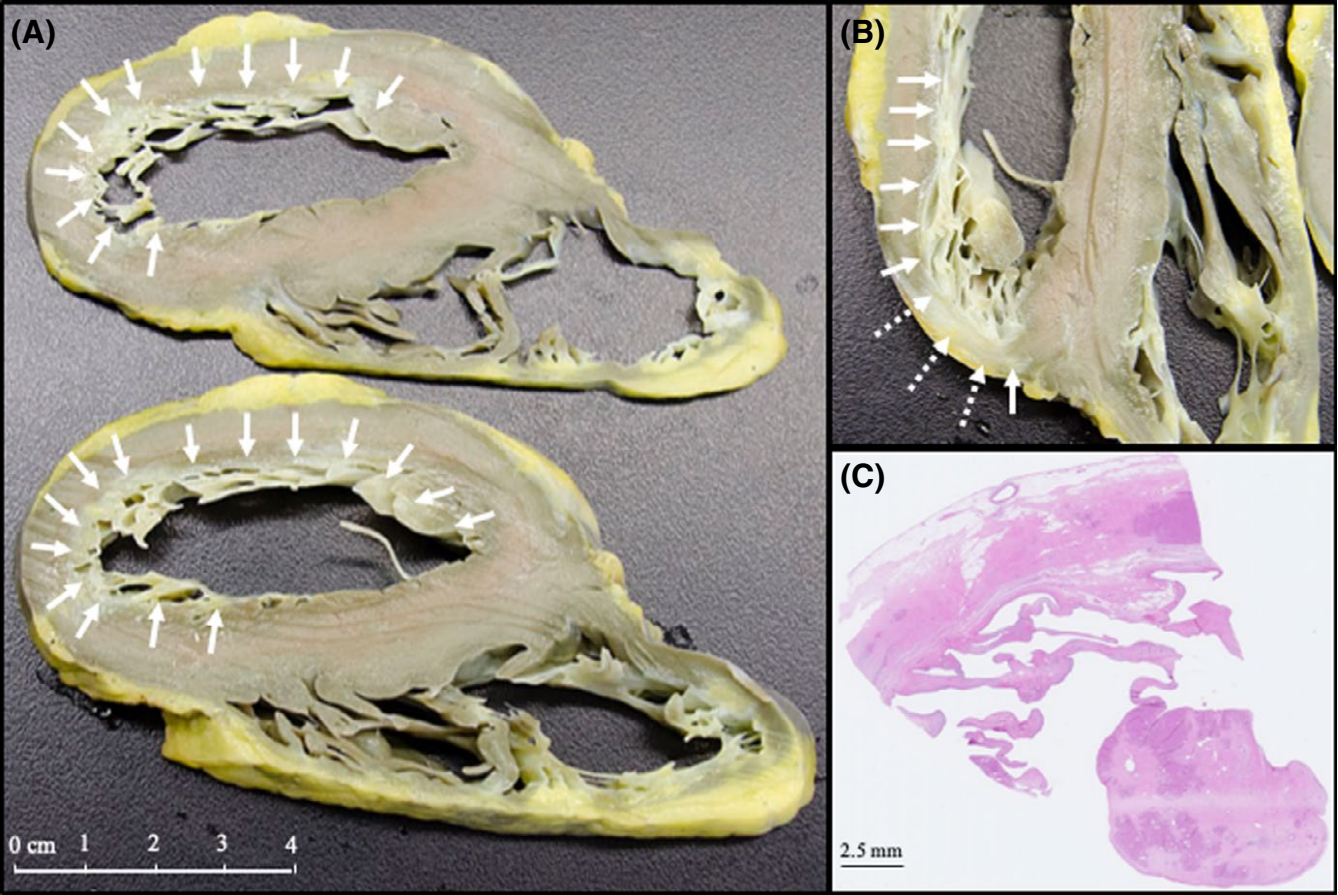
Panel A and B: Diffuse endocardial fibrosis (white arrows) affecting the inner half of both ventricles and sometimes affecting the full thickness of the myocardium (panel B, dashed arrows). Panel C: At low magnification, histology section shows that the myocardium and the posterior papillary muscle are almost completely replaced by dense fibrous tissue

Involvement of other organ systems by the disease was ruled out by computed tomography of the lung, gastroscopy with biopsy and also by quadriceps biopsy. Pulmonary function testing was normal with total lung capacity of 4.65 L (102% of predicted value), forced vital capacity of 2.65 L (92% of predicted value), forced expiratory volume in one second of 2.25 L (91% of predicted value), and DLCO 22 mL/mm Hg‐min (120% of predicted value). As the patient failed to improve with maximal medical therapy, including perfusion of Milrinone, in the absence of SSc lung involvement or pulmonary hypertension and limited manifestations in other organs attributed to SSc (esophageal reflux with normal biopsy, Raynaud phenomenon without digital ulcers), the patient was put on a transplantation list. After more than 6 months of intrahospital wait for a compatible heart, the patient underwent successful orthotopic heart transplant with usual perioperative anesthesia care. Of notice, the patient had normal pulmonary pressure on invasive monitoring. On the acute postoperative period, the patient suffered from acute tubular necrosis with a transient need for continuous venovenous hemofiltration. The patient recovered quickly from transplantation without significant clinical complication. Three weeks after the surgery, she was dismissed from the hospital. Four years after transplantation, she remains free of symptoms related to heart failure. She is able to walk 2‐3 km without dyspnea. The skin manifestation of SSc are greatly improved, with a MRSS of 7 out of 51, on standard postheart transplantation immunotherapy consisting of mycophenolate (360 mg PO BID) and tacrolimus (4 mg Po BID). Extensive fibrosis of the myocardium is showed in the explanted heart (Figure 2).

## 3 DISCUSSION

Heart transplantation is rarely considered in patients with SSc given the common significant concomitant organ involvement, including the lungs. Reports of isolated heart transplantation in these cases are scarce. In an extensive review of the literature, we found only 10 patients with predominant heart involvement consisting of severe left systolic dysfunction secondary with SSc who had undergone isolated heart transplantation.^1^, ^4^, ^5^, ^6^, ^7^ Of note, all patients with isolated heart transplantation had at least another systemic involvement, mostly gastrointestinal and/or musculoskeletal. Noteworthy, pulmonary involvement was not present in any patients. The mean age at time of transplantation was 24 ± 12 years old. At distance of at least one year from heart transplantation, 80% (n = 8) were alive at the time of their report, with good global status and no progression of SSc.

Systemic sclerosis is a connective tissue disease that affects primarily the skin. A study published in 1976 by Bulkley et al of Johns Hopkins Hospital included 52 cases of autopsies in which they found “contraction band necrosis and replacement of the myocardial tissue by patchy fibrosis, with no associated coronary artery disease or pulmonary hypertension”.^6^ Another study demonstrated that severe cardiac involvement was significantly more common in patients with rapid skin disease progression. The latter was associated with severe heart disease in 3% of the patients versus 1% of those with slow progression.^8^ Magnetic resonance imaging is an accurate and reliable technique for the diagnosis of cardiac involvement in SSc. As it is noninvasive, quantitative and highly sensitive, CMR appears to be the method of choice for the quantification of myocardial fibrosis that is usually subendocardial.^9^ Latest improvements in CMR techniques, like T1 mapping and extracellular volume fraction, reliably identify myocardial fibrosis and inflammation in SSc patients.^10^, ^11^, ^12^ Systemic sclerosis patients with symptomatic cardiac involvement have a poor prognosis, with 2‐year mortality rates of 60% and 5‐year mortality rates of 75%. Clearly, in presence of diffuse biventricular fibrosis demonstrated by CMR, significant clinical improvement and positive left ventricular remodeling is improbable. Patients with advanced myocardial fibrosis and left ventricular dysfunction with severe pulmonary involvement may be candidates for heart‐lung transplantation. While lung transplantation is not novel in SSc, isolated heart transplantation is rare. This is likely related to frequent involvement of other organs in SSc patients with cardiac manifestations, making them poor transplant candidates.^13^ Pulmonary involvement is common in patients with SSc and most often comprises fibrosis, interstitial lung disease, and/or pulmonary vascular disease leading to arterial pulmonary hypertension. Manifestations of interstitial lung disease in SSc predominantly affect the interstitium and pulmonary vasculature. The introduction of high‐resolution CT scan has significantly increased the sensitivity of radiographic diagnosis. The extreme sensitivity of high‐resolution CT scan makes it easy to identify evidence of fibrosis. Such changes are seen in 55%‐65% of patients with SSc, including up to 96% of those with abnormal pulmonary function test.^14^

Pulmonary function test, high‐resolution pulmonary CT scan, and right‐side cardiac catheterization are mandatory in the evaluation of patients with systemic sclerosis when isolated heart transplantation is contemplated to rule out any significant pulmonary involvement. Lung transplant is an accepted treatment option for SSc patients with significant pulmonary fibrosis and no evidence of other organ dysfunction. The physiopathology of heart involvement in systemic sclerosis is that various susceptibility and precipitant factors promote immune system activation with subsequent systemic vascular inflammation.^15^ The latter leads to chronic microvascular damages and subsequent myocardial fibrosis.

The role of cardiac transplantation in the patient with no pulmonary involvement but severe cardiac dysfunction secondary to SSc appears to be an interesting and valuable option.^5^ Moreover, as demonstrated in our case, chronic immunosuppression after heart transplantation benefits to the control of systemic sclerosis severity. In light of the favorable evolution of our patient and similar outcomes in other patients with SSc and isolated heart transplantation,^1^, ^4^, ^5^, ^6^, ^7^ we suggest that heart transplantation is relatively safe and lifesaving procedure in carefully chosen SSc patients with primary cardiac involvement.

## CONFLICT OF INTEREST

None declared.

## AUTHORS’ CONTRIBUTIONS

FTG, PYT, and MS: reviewed the literature and prepared the manuscript. MS, AI, JB, and PV: provided care for the patient during hospitalizations and follow‐up. PJ: proceeded to pathologic specimens analysis and was implicated as anatomopathology consultant. PYT, MD, and MS: provided feedback on the manuscript with further adjustments. All authors read and approved the final manuscript.
